# Allele-Specific RNA Silencing of Mutant Ataxin-3 Mediates Neuroprotection in a Rat Model of Machado-Joseph Disease

**DOI:** 10.1371/journal.pone.0003341

**Published:** 2008-10-08

**Authors:** Sandro Alves, Isabel Nascimento-Ferreira, Gwennaëlle Auregan, Raymonde Hassig, Noëlle Dufour, Emmanuel Brouillet, Maria C. Pedroso de Lima, Philippe Hantraye, Luís Pereira de Almeida, Nicole Déglon

**Affiliations:** 1 Center for Neurosciences and Cell Biology, University of Coimbra, Coimbra, Portugal; 2 Faculty of Pharmacy, University of Coimbra, Coimbra, Portugal; 3 Faculty of Sciences, University of Coimbra, Coimbra, Portugal; 4 CEA, Institute of Molecular Imaging (I2BM) and Molecular Imaging Research Center (MIRCen), Orsay, France; 5 CNRS URA 2210, Orsay, France; National Institutes of Health, United States of America

## Abstract

Recent studies have demonstrated that RNAi is a promising approach for treating autosomal dominant disorders. However, discrimination between wild-type and mutant transcripts is essential, to preserve wild-type expression and function. A single nucleotide polymorphism (SNP) is present in more than 70% of patients with Machado-Joseph disease (MJD). We investigated whether this SNP could be used to inactivate mutant ataxin-3 selectively. Lentiviral-mediated silencing of mutant human ataxin-3 was demonstrated *in vitro* and in a rat model of MJD *in vivo*. The allele-specific silencing of ataxin-3 significantly decreased the severity of the neuropathological abnormalities associated with MJD. These data demonstrate that RNAi has potential for use in MJD treatment and constitute the first proof-of-principle for allele-specific silencing in the central nervous system.

## Introduction

Machado-Joseph disease (MJD) or spinocerebellar ataxia type 3 (SCA3) is a dominantly inherited disorder of the central nervous system (CNS) characterized by a wide range of clinical symptoms, including gait and limb ataxia, peripheral neuropathy, bulging eyes, ophthalmoplegia, postural instability, dystonia, amyotrophy, dysarthria, nystagmus, lingual fasciculations, facial myokymia [1] and, in some cases, parkinsonism [2]. The neuropathological features of the disease involve the afferent and efferent cerebellar systems, *substantia nigra*, and cranial nerve motor nuclei [1] but recent evidence suggests that the *striatum* is also affected [3], [4]. Although initially identified in subjects of Portuguese Azorean descent [1], MJD is now the most common ataxia worldwide [5]. It is caused by an unstable CAG expansion in the coding region of the *MJD1* gene encoding the ataxin-3 (ATX3) protein [6]. The expansion in the mutant allele ranges from 55 to 86 CAG repeats and the length of the polyglutamine tract is inversely correlated with age at onset of the disease [7], [8]. The CAG expansion confers a toxic gain-of-function on the mutant protein, leading to the formation of neuronal intranuclear inclusions (NIIs) [9]. There is currently no available treatment.

Gene silencing by RNA interference (RNAi) has been successfully used to downregulate the expression of mutant genes and rescue phenotypes in various autosomal dominant neurodegenerative diseases, including Huntington's disease (HD) [10]–[12], familial forms of amyotrophic lateral sclerosis (ALS) [13], [14] and spinocerebellar ataxia type 1 (SCA1) [15]. However, *in vivo* studies to date have been performed with siRNAs that do not discriminate between the wild-type and mutant alleles. The loss of function of wild-type ataxin-3, which has been shown to play a role in ubiquitin-mediated proteolysis [16], might be deleterious. Strategies based on the presence of a single nucleotide polymorphism (SNP) have been proposed to ensure discrimination between wild-type and mutant transcripts [17]. An SNP has been identified at the 3′ end of the CAG tract of the ataxin-3 gene. This SNP is in linkage disequilibrium with the disease-causing expansion [18], [19]. In most MJD patients, the mutant allele carries the C variant [20]. This feature provided us with an opportunity to develop and validate an allele-specific siRNA silencing strategy for the treatment of 70% of MJD patients. Similar approaches could potentially be applied to other neurodegenerative diseases.

In the present study, we used lentiviral vectors (LV) encoding short-hairpin RNAs (shRNAs) targeting this SNP, to downregulate mutant human ATX3 (MUT-ATX3) *in vivo* in a selective manner. We demonstrate the therapeutic efficacy and selectivity of this approach in a rat model of MJD [4].

## Results

**Figure 1:**
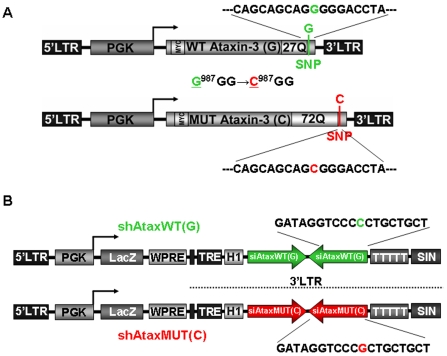
The single nucleotide polymorphism strategy used for the specific elimination of mutant or wild-type human ataxin-3 (ATX3) by RNA interference. A) Schematic representation of the lentiviral constructs encoding wild-type human ataxin-3 (27 CAG repeats) or mutant human ataxin-3 (72 CAG repeats) under control of the phosphoglycerate kinase-1 (PGK-1) promoter. Immediately after the last CAG repeat in the 3′ end, there is a linked single nucleotide polymorphism (SNP) (G ^987^GG→C ^987^GG) between wild-type and mutant human ataxin-3. B) Diagram of the shAtax vectors used to downregulate human ataxin-3: shRNA cassette under control of the H1 promoter (pol III) and a separate cassette containing the *lacZ* reporter gene under control of the PGK-1 promoter, making it possible to follow the expression of infected neurons. These shRNAs were designed to silence wild-type (shAtaxWT) or mutant human ataxin-3 (shAtaxMUT) selectively, making use of the (G ^987^GG→C ^987^GG) SNP.

We developed two lentiviral vectors encoding siRNAs targeting an SNP (G ^987^GG→C ^987^GG) located at the 3′ end of the CAG expansion of the ataxin-3 gene for the specific silencing of mutant human ataxin-3 mRNA but not of the wild-type ataxin-3 mRNA. The wild-type ataxin-3 gene has a G at position 987, whereas the mutant ataxin-3 has a C at this position. We therefore designed two siRNAs corresponding to this SNP. The sequences are identical with the exception of a single nucleotide (G/C polymorphism) at the center of the shRNA molecule: shAtaxMUT(C) and shAtaxWT(G) (Fig. 1A). These shRNAs were inserted in the 3'LTR of a lentiviral vector containing the H1 promoter. The *lacZ* reporter gene was inserted into these constructs downstream from the internal mouse phosphoglycerate kinase 1 (PGK) promoter, to facilitate the identification of transduced cells (Fig. 1B).

### Selectivity and efficacy of mutant and wild-type ataxin-3 silencing *in vitro*

**Figure 2:**
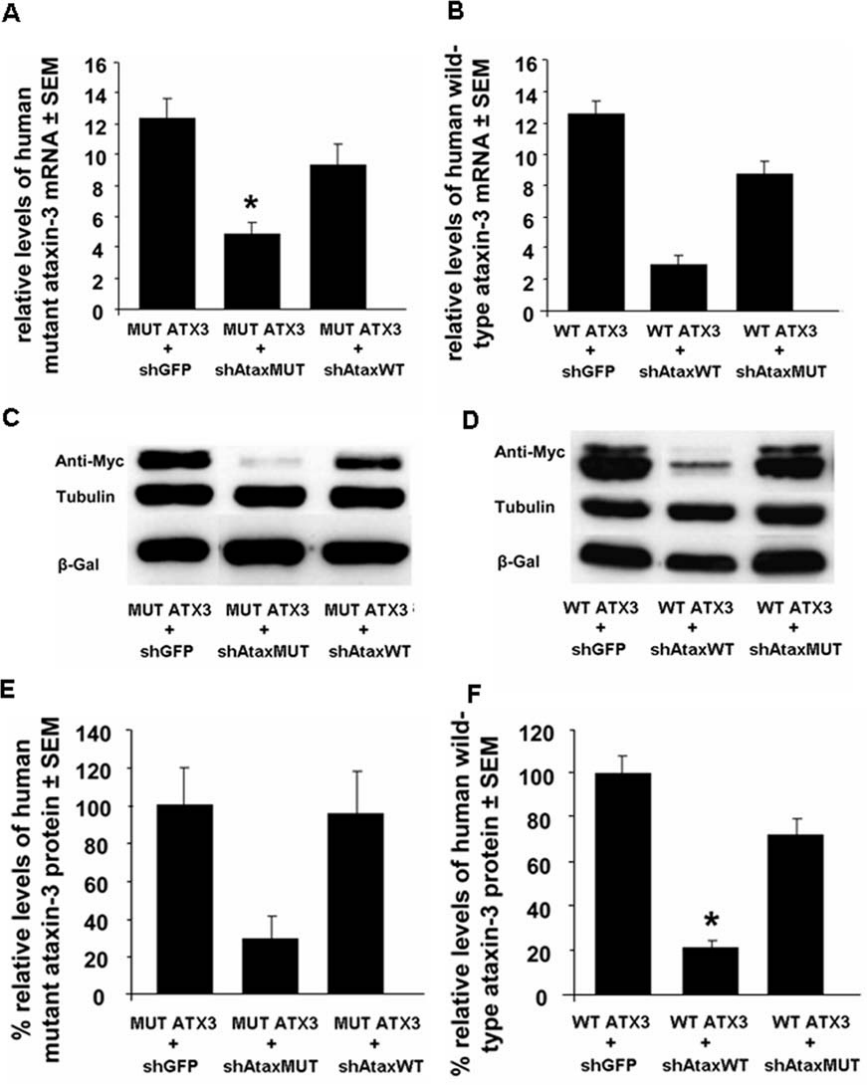
shRNAs mediate the *in vitro* allele-specific suppression of mutant or wild-type human ataxin-3 by RNAi. A–F) shAtaxMUT- or shAtaxWT-encoding plasmids selectively targeting mutant ataxin-3 and wild-type ataxin-3, respectively, resulted in much lower levels of these proteins than the mistargeted control (shGFP) or the non allele-specific shRNA. Quantitative real-time PCR analysis showing the silencing of human ATX3 mRNA in 293T cells co-expressing mutant human ataxin-3 (MUT ATX3) (A, top left) or wild-type human ataxin-3 (WT ATX3) (B, top right) and shAtaxWT, shAtaxMUT, or shGFP. Endogenous ß-actin mRNA was used as an internal control for the normalization and quantitative analysis of the ataxin-3 mRNA levels. Results are expressed as the mean elative mRNA level±SEM. C and D) Western-blot analysis of lysates of 293T cells co-transfected with the plasmid constructs encoding MUT ATX3 (C, middle left) or WT ATX3 (D, middle right) and the shAtax vectors (48 hours after calcium phosphate-mediated transfection; ratio ATX3/shRNA 1∶5). Tubulin staining is shown as a loading control. E and F) Optical densitometry was normalized according to the amount of tubulin loaded in the corresponding lane. A partition ratio was calculated and expressed as a percentage (bottom). All western blots and RT-PCRs shown are representative of three or four independent experiments. Statistical significance was evaluated using Fisher's test (*p<0.05).

Quantitative RT-PCR analysis of 293T cells co-transfected with the shAtaxMUT(C) vector and a vector expressing the human mutant ataxin-3(C) gene (Fig. 1B) demonstrated robust mRNA degradation (Fig 2A). By contrast, ataxin-3 mRNA levels were slightly decreased by co-transfection with shAtaxMUT(C) and the wild-type ataxin-3(G) gene or with shAtaxWT(G) and the mutant ataxin-3(C) (Fig. 2A–B), demonstrating the selectivity of the silencing. As a control, we included an siRNA (shGFP) targeting the green fluorescent protein (Fig. 2A). RT-PCR with oligomers targeting *lacZ* confirmed that the decrease in ataxin-3 mRNA levels reflected siRNA efficiency rather than variations in transfection efficiency (data not shown).

We then investigated whether this silencing was associated with a decrease in wild-type and mutant ataxin-3 protein levels. Co-transfection with the mutant ataxin-3 plasmid and the specific shAtaxMUT led to significant (∼70%) downregulation, as observed on western blots, whereas co-transfection with the non specific shAtaxWT had limited effect on mutant ataxin-3 protein levels (Fig. 2C and E). Conversely, as expected, strong down-regulation of wild-type ataxin-3(G) expression was observed in the presence of shAtaxWT(G), whereas shAtaxMUT(C) had a limited effect on wild-type ataxin-3(G) expression (Fig. 2D and F).

**Figure 3:**
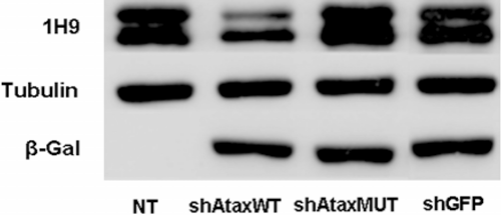
shRNA-expressing plasmids mediate the allele-specific silencing of endogenous wild-type ataxin-3 in transiently transfected human 293T cells. A) Western blot of human 293T cells transfected with different shRNA (shAtaxWT, shAtaxMUT and shGFP)-expressing plasmids (5 µg; 48 h post-transfection). The blot clearly indicates that shAtaxWT downregulates endogenous/wild-type human ATX3, whereas shAtaxMUT has no silencing effect. shGFP was used as a control vector and the protein tubulin was used as a loading control. This western blot is representative of three independent experiments.

We further assessed the functionality and efficacy of our siRNA in a more physiological situation, by analyzing the silencing of endogenous ataxin-3 in human embryonic kidney 293T cells. We transfected human 293T cells with the shAtaxWT(G), shAtaxMUT(C) or shGFP plasmid. The level of endogenous wild-type ataxin-3 was determined by western blotting and found to be lowered by transfection with shAtaxWT(G), but not with shAtaxMUT(C) or shGFP (Fig. 3). These data demonstrate the efficacy and selectivity of allele-specific ataxin-3 silencing.

### Selectivity and efficacy of mutant ataxin-3 silencing in rat brain

No data are available concerning the allele-specific silencing of polyglutamine disorders *in vivo*. We assessed the efficacy and selectivity of this approach in the recently developed rat model of MJD [4]. The injection of lentiviral vectors encoding mutant ataxin-3 into the brain of adult rats leads to the appearance of disease reproducing the key characteristics of MJD. No such disease was induced by the injection of wild-type full-length ataxin-3. Misfolded mutant ataxin-3 proteins are detected within one week of infection and these ubiquitin-positive inclusions progressively accumulate in the nucleus of infected cells. Neuronal dysfunction, with the presence of pycnotic nuclei and a loss of expression of neuronal markers, is observed in the *substantia nigra* (TH, VMAT2) and *striatum* (DARPP-32, NeuN, TH) two months after injection, in this model.

We assessed the efficacy and selectivity of allele-specific silencing, by injecting lentiviral vectors encoding human mutant ataxin-3(C), together with the corresponding shRNA (shAtaxMUT(C)), into rat *striatum*. We recently demonstrated that the *striatum* is affected in MJD, whether in our rat model, transgenic mice or patients [4]. As a control, animals were injected with human mutant ataxin-3 and shAtaxWT(G) or shGFP. Three weeks after injection, two animals per group were killed to evaluate expression of the mutant ataxin-3 gene and of the *lacZ* reporter gene present in the vector encoding the small hairpin RNA.

**Figure 4:**
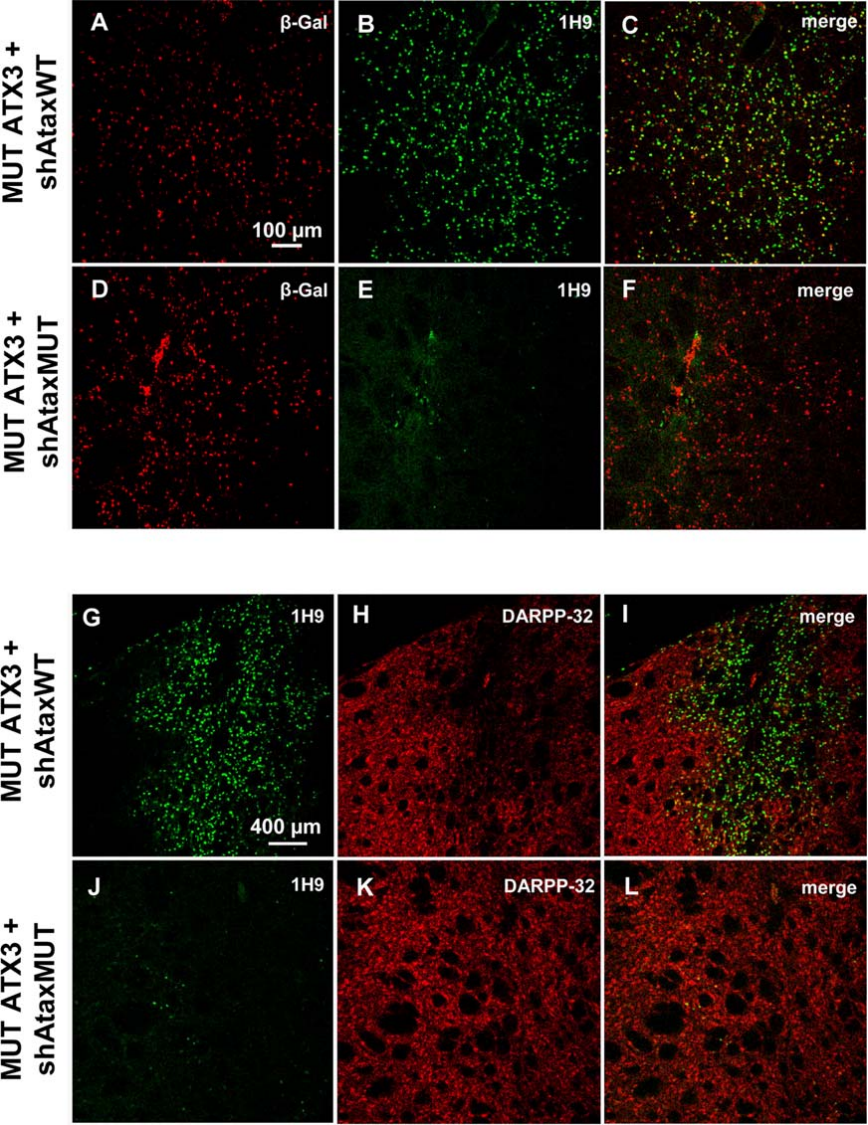
Efficient allele-specific suppression of mutant human ataxin-3 in the rat brain at an early time point (3 weeks) mediates striatal neuroprotection. A–L) Laser confocal microscopy showing the effects of recombinant lentiviral vectors encoding shAtaxMUT or shAtaxWT and MUT ATX3 in the rat brain striatum at an early time point (3 weeks). β-galactosidase, expressed from a separate PGK*-lacZ* cassette in the vectors, allows identification of infected neurons (A and D). shAtaxMUT specifically silences MUT ATX3, promoting the clearance of MUT ATX3-positive aggregates (E and J), whereas shAtaxWT has almost no effect on MUT ATX3 expression (B and G). A considerable loss of DARPP-32-immunoreactivity is observed in rat striatum co-infected with MUT ATX3 and shAtaxWT (H and the merged image I), whereas no DARPP-32 downregulation is observed in rat striatum co-infected with MUT ATX3 and shAtaxMUT (K and the merged image L), suggesting neuroprotection. The adult rats were co-injected bilaterally in the striatum with MUT ATX3 and the shAtaxWT or shAtaxMUT vectors (n = 2) and were killed three weeks later. All the pictures were taken around the injection site.

**Figure 5:**
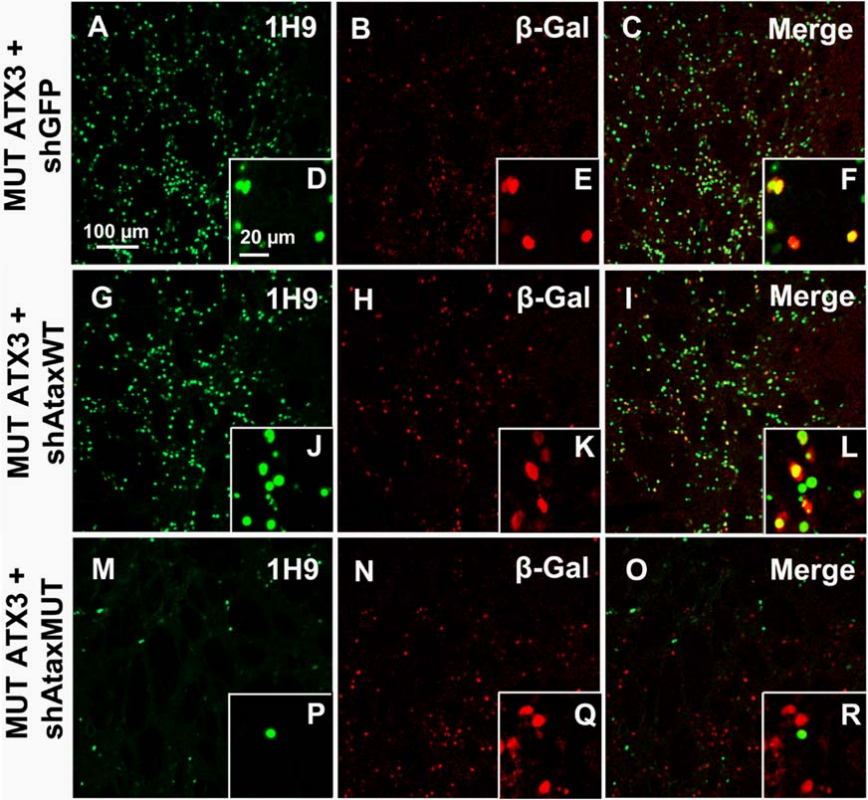
Allele-specific silencing of mutant human ataxin-3 in rat brain. A) Laser confocal microscopy, showing neuronal transduction 2 months after injection in the rat striatum with recombinant lentiviral vectors encoding shAtaxMUT (n = 7), shAtaxWT (n = 8) or shGFP (n = 4) and mutant human ataxin-3 (MUT ATX3). The viral vectors also contained a separate PGK-*LacZ* cassette encoding β-galactosidase, to facilitate the detection of infected neurons (B, H, N and E, K, Q, high magnification). In adult rats expressing MUT ATX3 and shAtaxMUT (n = 7), the number of neurons containing MUT ATX3-positive aggregates was much smaller (M) and the high magnification merged image (R) indicates that the few cells positive for MUT ATX3 did not express the *lacZ* reporter gene present in the shAtaxMUT vector. These cells were therefore not transduced with the vectors encoding the silencing sequences. By contrast, in animals expressing MUT ATX3 and shGFP (n = 4) or the control shAtaxWT (n = 8) (A and G, respectively) high magnification merged images show many MUT ATX3-positive cells simultaneously expressing the *lacZ* reporter gene present in both shAtaxWT or shGFP (F and L, respectively). The figure shows representative images of immunohistochemical stainings that were reproducible among the different groups.

In animals infected with lentiviral vectors encoding mutant ataxin-3(C) and the non specific shAtaxWT(G) or the shGFP control, immunohistochemical analysis of coronal rat brain sections with anti-ataxin-3 (1H9) and β-galactosidase antibodies showed that many neurons expressed both transgenes (Fig. 4A–C, Fig 5A–L). In animals expressing mutant ataxin-3 and shAtaxMUT(C), far fewer neurons produced the pathogenic protein (Fig. 4E, Fig. 5M). The merged images (Fig. 4F, Fig. 5O) indicate that only a few mutant ataxin-3-positive-cells did not express the *lacZ* reporter gene present in the shAtaxMUT(C) vector (high magnification Fig. 5R). These cells corresponded to neurons not co-infected with both vectors, which were therefore not treated with the siRNA.

**Figure 6:**
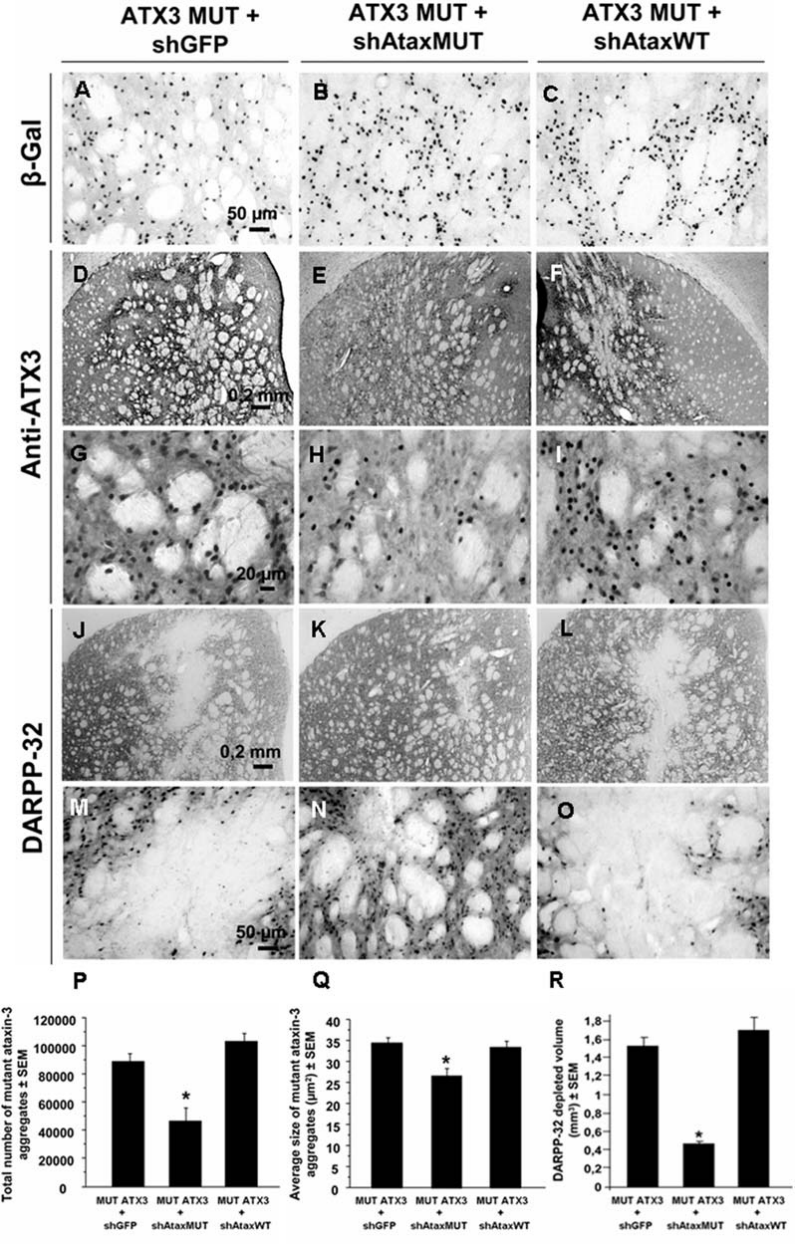
Allele-specific silencing of mutant human ataxin-3 mediates robust reduction of the number of ataxin-3 inclusions and preservation of DARPP-32 immunoreactivity in the rat striatum. A–R) Co-overexpression of MUT ATX3 and various shRNAs (shAtaxWT, n = 8; shAtaxMUT, n = 7 and shGFP, n = 4) in the striatum of adult rats, 2 months post-injection. The vectors encoding the shRNA cassette and the *lacZ* reporter gene infect an extensive region of the rat striatum, as shown by β-galactosidase immunoreactivity (A, B and C). shAtaxMUT specifically downregulates MUT ATX3, promoting a significant decrease in the number of MUT ATX3-positive aggregates (E and H ), whereas shAtaxWT has almost no effect on MUT ATX3 expression (F and I), as shown by comparison with the results obtained with the mistargeted shGFP (D and G). A major loss of DARPP-32 immunoreactivity is observed in the striatum infected with MUT ATX3 and shAtaxWT (L and O) or shGFP (J and M), whereas minor DARPP-32 is observed in the striatum infected with shAtaxMUT (K and N), this downregulation being limited to the needle track area. P–R) Quantification of the effect of the different shRNAs on the absolute number (P) and mean size/surface* (Q) of MUT ATX3-positive cells (*p<0,05). R) Quantitative analysis of the DARPP-32-depleted region in the brains of rats in which the striatum was injected with MUT ATX3 and various shRNAs. The lesion volume in brains infected with shAtaxMUT and MUT ATX3 is much smaller than that in brains infected with shAtaxWT or shGFP, indicative of a neuroprotective effect conferred by the selective shAtaxMUT. Statistical significance was evaluated with Fisher's test. (* the mean size of the objects was estimated taking into account the pixels with a gray-scale level for intensity below the mean value, used as a threshold).

**Figure 7:**
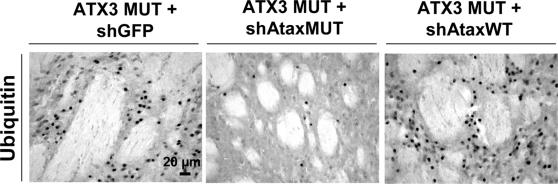
Reduction of ubiquitin-positive inclusions in the striatum of adult rats as result of mutant human ataxin-3 knock-down. Animals infected with MUT ATX3 and the control shGFP (left; n = 4) or shAtaxWT (right, n = 8) show the accumulation of ubiquitin-positive inclusions, typical biomarkers of neuropathology, whereas no such accumulation is observed in animals co-infected with MUT ATX3 and the selective shAtaxMUT (middle, n = 7). The figure shows representative images of ubiquitin immunohistochemical stainings that were reproducible among the different groups.

**Figure 8:**
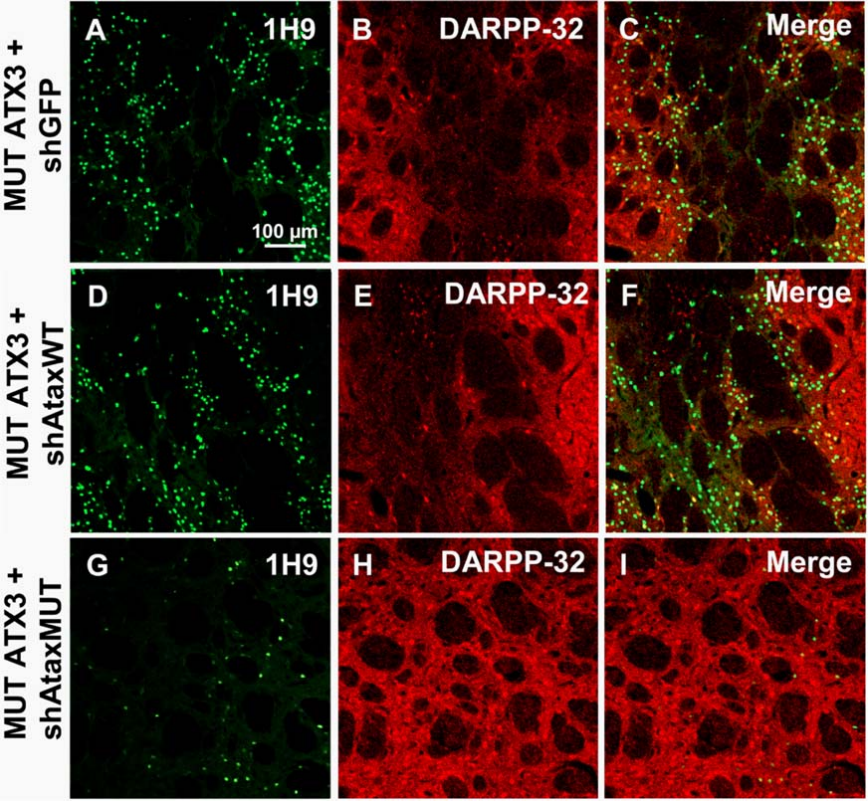
Rescue of DARPP-32 immunoreactivity and reduced accumulation of ataxin-3 inclusions upon shAtaxMUT expression. A–I) Laser confocal microscopy showing the expression of recombinant lentiviral vectors expressing MUT ATX3 and shGFP (n = 4), shAtaxWT (n = 8) or shMUT (n = 7) and its effects on DARPP-32 expression in the rat striatum 2 months post-injection. Slight DARPP-32 downregulation is observed in striatum infected with MUT ATX3 and the selective shAtaxMUT (H and the merged image I), whereas a significant loss of DARPP-32-immunoreactive neurons is observed in rat striatum co-infected with MUT ATX3 and shAtaxWT (E and the merged image F) or MUT ATX3 and the control shGFP (B and the merged image C). The figure shows representative images of immunohistochemical stainings that were reproducible among the different groups.

We assessed the efficacy of this approach further, by carrying out a, histological evaluation at two months, at which time MJD pathology was severe [4]. β-galactosidase staining indicated a similar transduction efficiency for all groups eight weeks post-injection (Fig. 6A–C). In animals injected with mutant ataxin-3, we observed a typical accumulation of inclusions (Fig. 6D, G, P), with a mean size of 34.3±1.4 µm^2^ (Fig. 6Q) and a loss of DARPP-32 production (Fig. 6R). Comparison between this group and that including animals treated with the non selective siRNA (shAtaxWT(G); Fig. 6C, F, I, L, O) revealed no statistically significant differences in terms of the formation of inclusions (Fig. 6P, Q) and the DARPP-32-depleted area (Fig. 6R). Co-transduction with mutant ataxin-3(C) and shAtaxMUT(C) significantly decreased the number of inclusions (48.2±10.8 % considering shGFP as the control; 55.5%±9.3% considering shAtaxWT as the control) and the apparent size of the remaining inclusions (by 12.7±4.3% if compared with shGFP and 9.92±4.4 % if compared with shAtaxWT(G); Fig. 6P–R). Similar results were obtained with ubiquitin staining, which also showed important reduction in the number of inclusions upon mutant ataxin-3 silencing with shAtaxMUT (Fig. 7). Double staining for DARPP-32 and ataxin-3 showed co-localization between ataxin-3 inclusions and DARPP-32 loss of immunoreactivity in control animals (Fig. 8A–F), and rescue of DARPP-32 immunoreactivity co-localizing with reduction of inclusion number upon shAtaxMUT expression (Fig. 8G–I).

**Figure 9:**
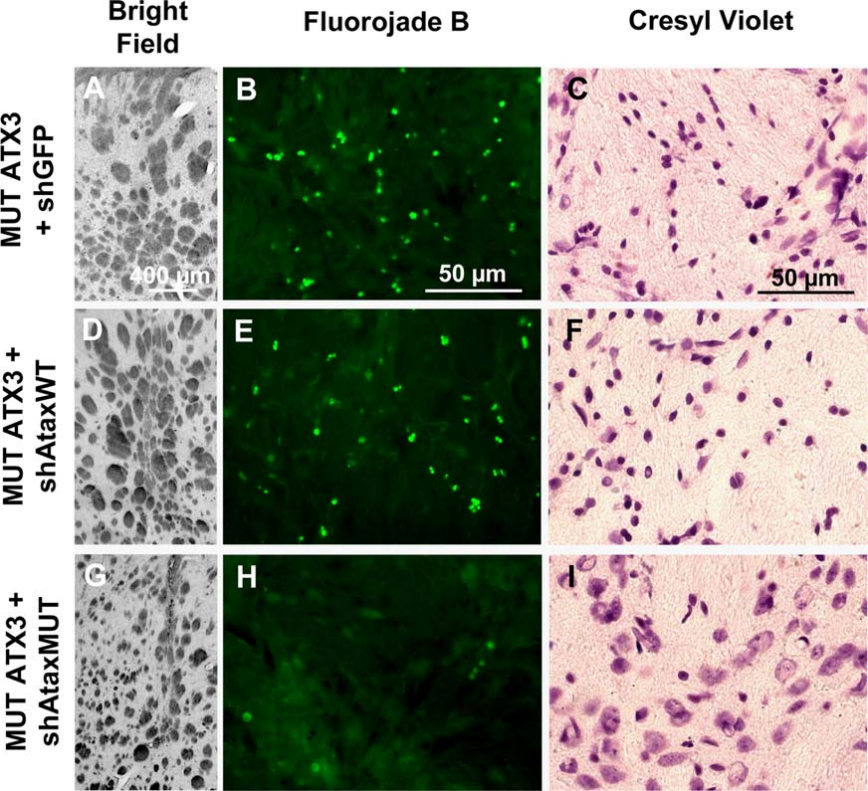
Allele-specific silencing of mutant human ataxin-3 prevents neurodegeneration in the adult rat striatum. Coalescence of the internal capsule of the striatum is observed after co-infection with MUT ATX3 and shGFP (A, n = 4) or shAtaxWT (D, n = 8), at 2 months, on a bright-field photomicrograph, whereas no signs of striatal shrinkage are observed following co-infection with MUT ATX3 and the specific shAtaxMUT (G, n = 7) (left column). Neurodegeneration in rats co-infected with MUT ATX3 and shGFP (B) or shAtaxWT (E) is observed two months after injection on Fluorojade B-stained sections, but not in rats co-infected with MUT ATX3 and the selective shAtaxMUT (H) (middle column). Pycnotic nuclei are visible on cresyl violet-stained sections, suggesting cell injury and striatal degeneration after brain co-infection with MUT ATX3 and the control shGFP (C) or the non specific shAtaxWT (F), in adult rats at 2 months after injection. No such nuclei are observed on sections from rats co-infected with MUT ATX3 and the specific shAtaxMUT (I) (right column). All the pictures were taken around the injection site area and show representative immunohistochemical stainings that were reproducible among the different groups.

Finally, the staining of degenerating neurons with fluorojade B (Figure 9B, E, H) and cresyl violet (Figure 9C, F, I) further demonstrated that the selective silencing of mutant ataxin-3 markedly decreased the number of degenerating neurons and atrophic nuclei, leading to typical striatum shrinkage (Fig. 9A, D, G).

Thus, allele-specific silencing efficiently and selectively inhibits human mutant ataxin-3 production, greatly decreasing the formation of disease-associated inclusions and neuronal dysfunction *in vivo*.

## Discussion

We show here that allele-specific silencing of human mutant ataxin-3 is effective and selective *in vivo*. Autosomal dominant CNS diseases are good candidates for RNAi therapy, and Machado-Joseph disease is of particular interest because the presence of an SNP is linked to the disease. Other potential disorders suitable for allele-specific treatment include dystonia, in which a three-base pair deletion has been identified in the mutant torsinA gene product [21] and point mutations in the superoxide dismutase gene have been implicated in familial forms of amyotrophic lateral sclerosis (ALS) [22]. These diseases provide unique opportunities to silence the mutant gene product while preserving the wild-type protein.

It has been shown *in vitro* that targeting the CAG tract of ataxin-3 abolishes the expression not only of the mutant allele, but also of the wild-type allele [17]. Alternatively, the mutant MJD allele could be specifically targeted by making use of three single nucleotide polymorphisms present in the human population: C^987^GG/G^987^GG (Arg to Gly), A^669^TG/G^669^TG (Met to Val) and TAA^1118^/TAC^1118^ (Stop to Tyr). Analysis of these polymorphic alleles showed there to be eight different haplotypes. However, the A^669^-C^987^-A^1118^ haplotype was found in 72% of MJD patients from 249 families from different countries, but in only 2% of the normal population [20]. This allele is present in about half of all Portuguese patients [20] and is associated exclusively (100%) with mutant polyQ expansions in Japanese MJD patients [23]. It may therefore be possible to treat a large proportion of MJD patients with a single shRNA specific for the mutant allele.

We developed two lentiviral vectors encoding previously described shRNA sequences [17] specific for the C^987^GG (MUT) or G^987^GG (WT) alleles. These shRNAs were not produced according to available rules for the design of optimal siRNAs [24]. Instead, their design was based on the presence of the polymorphism, this polymorphic sequence being placed at the center of the shRNA. These shRNAs had been shown to function correctly *in vitro*, but not within the context of lentiviral vectors (24). It was therefore difficult to predict the results, particularly *in vivo*. Our data clearly demonstrate that shAtaxMUT selectively and efficiently silences the human mutant ataxin-3 gene. *In vivo*, we observed a significant decrease in the formation and accumulation of ataxin-3-positive aggregates (∼50%) and preservation of DARPP-32 neuronal marker expression (∼70%).

The selectivity of the approach was demonstrated *in vitro*, by the preservation of wild-type ataxin-3 expression upon shAtaxMUT co-expression, and *in vivo*, by the limited effect of shAtaxWT on mutant ataxin-3 gene expression. It should be noted that shAtaxMUT and shAtaxWT recognize only human ataxin-3 mRNAs, as the targeted region displays limited similarity of sequence to the endogenous rat ataxin-3 messenger RNA. These experiments therefore replicate allele-specific silencing as it would occur in human patients, with the preservation of wild-type ataxin-3 gene expression, an important safety measure given the role of ataxin-3 in proteolysis.

ATAX3 has been shown to act as a polyubiquitin-binding protein, linking its normal function to protein surveillance pathways [25]. Ataxin-3 may recruit polyubiquitinated substrates through its UIM domains, promoting cleavage via its Josephin domain, leading to proteasomal degradation [26]–[28] The potential role of ATAX3 in proteolysis might, upon silencing, impair neuronal functions and viability. No particular phenotype of neuronal degeneration was observed in MJD knockout (KO) animals, but an increase in protein ubiquitination was reported [29]. Significant dysregulation of genes involved in the ubiquitin-proteasome pathway, structure/motility, and signal transduction have been reported in KO *C. elegans* [30]. Furthermore, ataxin-3 silencing may be well tolerated in wild-type animals, whereas similar silencing in the context of MJD may have unfavorable effects on disease progression. Studies in *Drosophila* have shown that wild-type ataxin-3 decreases the neurotoxicity of mutant ataxin-3 via a mechanism involving ubiquitin and the proteasome [31]. This protective role of wild-type ataxin-3 in MJD would not be perceptible in a non pathological context. Consistent with these findings, homozygous SCA3 patients with two mutant alleles present a more severe disease phenotype than patients with a single mutant allele [32], [33]. It remains unclear whether adult neurons can tolerate partial non selective endogenous ataxin-3 downregulation. As a precautionary measure, it is therefore advisable to try to preserve wild-type ataxin-3 expression in gene silencing approaches.

Our data suggest that the use of an allele-specific shRNA efficiently decreases both mutant ataxin-3 production and MJD-associated neuropathological symptoms. A recent report implicated RNA toxicity in MJD pathology, providing further support for treatment at RNA level [34]. However, the clinical development of siRNA-based treatments for MJD still faces several hurdles. Studies are currently underway to demonstrate the safety and long-term efficacy of the approach, to determine which brain areas should be targeted for therapeutic benefit, and to validate a delivery system for administration to large areas of the brain. The recent incorporation of lentiviral vectors into clinical practice for the treatment of Parkinson's disease has, however, opened up new opportunities for the potential treatment of fatal and incurable diseases such as MJD.

In conclusion, we have generated lentiviral vectors markedly improving MJD-associated neuropathological signs *in vivo*. These data suggest that it is feasible to treat MJD patients by the selective knockdown of mutant ataxin-3.

## Materials And Methods

### Engineering of shRNAs plasmids

We used two shRNAs discriminating between wild-type and mutant human ataxin-3 mRNA molecules. We engineered a small hairpin RNA specifically targeting the single nucleotide polymorphism (G ^987^GG→C ^987^GG). A shRNA targeting the Green Fluorescent Protein (shGFP) was used as a control. The sequences of the oligomers used were as follows: **shAtaxMUT**(CTAGTTTCCAAAAAAGCAGCAGC ^987^GGGACCTATCTCTCTTGAAGATAGGTCCCGCTGCTGCTGGGGATCTGTGGTCTCATACAGAAC); **shAtaxWT**(CTAGTTTCCAAAAAAGCAGCAGG ^987^GGGACCTATCTCTCTTGAAGATAGGTCCCCCTGCTGCTGGGGATCTGTGGTCTCATACAGAAC); **shGFP**(CTAGTTTCCAAAAAAAGCTGACCCTGAAGTTCATCTCTTGAATGAACTTCAGGGTCAGCTTGGGGATCTGTGGTCTCATACAGAAC). Each of these oligomers and the primer H1-3F (CACCGAACGCTGACGTCATCAACCCG) were used for PCR with the pBC-H1 plasmid (pBC plasmid; Stratagene, Amsterdam, The Netherlands) containing the H1 promoter (Genbank: X16612, nucleotides 146–366) as a template. The PCR product was inserted into the pENTR/D-TOPO vector (Invitrogen, Cergy Pontoise, France). The H1-shRNA cassette was then transferred, with the LR clonase recombination system, into the SIN-cPPT-PGK-nls-LacZ-LTR-TRE gateway vector (SIN-CWP-nlsLacZ-TRE-Gateway), which contains a tetracycline-regulated operator upstream from the H1 promoter in the 3′LTR. A LacZ reporter gene was also inserted downstream from the PGK promoter, facilitating the identification of infected neurons.

### Lentiviral vector production

Lentiviral vectors encoding the various shRNAs and human full-length wild-type(G) (27Q) or mutant ataxin-3(C) (72Q) [4] were produced in 293T cells, with a four-plasmid system, as previously described [35]. The lentiviral particles were resuspended in 1% bovine serum albumin (BSA) in phosphate-buffered saline (PBS). The viral particle content of batches was determined by assessing HIV-1 p24 antigen levels (RETROtek, Gentaur, Paris, France). Viral stocks were stored at −80°C until use.

#### Cell culture and transfection

HEK 293T cells were cultured in DMEM (Gibco, Paisley, Scotland, UK) supplemented with 10% fetal bovine serum (FBS, Gibco, Paisley, Scotland, UK), 2 mM L-glutamine, 4500 mg/l glucose, 25 mM HEPES, 100 U/ml penicillin, and 100 U/ml streptomycin (Gibco, Paisley, Scotland, UK) at 37°C in a 5% CO_2_/95% air atmosphere. On the day before transfection, 293T cells were plated in six-well tissue culture dishes (Costar, NY, USA) at a density of 700,000 cells per well. The cells were co-transfected by the calcium phosphate method, with SIN-W-PGK-ATX3 72Q (mutant human ataxin-3, 1 µg) or SIN-PGK-W-ATX3 27Q (wild-type ataxin-3, 1 µg) and shATX3 or shGFP (5 µg). Six hours later, the medium was removed and replaced with fresh medium. Forty-eight hours after transfection, the cell cultures were washed with cold PBS, treated with trypsin and the cells were collected by centrifugation.

#### Western blotting

Cells were collected by centrifugation (1000×*g*, 10 min). The pellets were incubated on ice in lysis buffer (150 mM NaCl, 50 mM Tris-base, pH 7.4, 5 mM EDTA, 1% Triton and 0.5% protease inhibitor cocktail; Sigma) for 30 minutes, with vortexing every 10 minutes. Homogenates were centrifuged at 13,000×*g* for 30 minutes at 4°C and supernatants were stored at −80°C. Protein concentration was determined with the Bradford protein assay (BioRad, Munich, Germany). Protein extract (20 µg) was resolved by electrophoresis in a 7.5 % or 12% SDS-polyacrylamide gels. The proteins were transferred onto nitrocellulose membranes (Schleicher & Schuell Bioscience, Germany) in TG 10×liquid concentrate buffer (Amresco, Ohio, USA) containing 192 mM glycine, 25 mM Tris-HCl, and 20% methanol. The membranes were blocked by incubation in 5% nonfat milk powder in 0.1% Tween 20 in Tris-buffered saline (T-tris-buffered saline) for 1 h at room temperature, and were then incubated overnight with the following primary antibodies diluted in blocking buffer: anti-Myc tag antibody clone 4A6 (1∶1000, Upstate, Cell Signaling Solutions, NY, USA), anti-β-galactosidase antibody (1∶4000; Chemicon, Temecula, CA,USA) and anti-tubulin antibody (1∶4000, Sigma, Saint Louis, MO, USA). Blots were washed three times in Tris-buffered saline containing 0.1% Tween 20 (TBS-T), for 20 min each, and incubated with a horseradish peroxidase (HRP)-coupled secondary biotinylated goat anti-mouse or anti-rabbit IgG antibody (1∶5000; Vector Laboratories, Burlingame, CA) for 1 h at room temperature. Blots were washed three times in TBS-T, for 20 minutes each, and binding to antigens was detected by enhanced chemiluminescence (ECL+, Amersham Pharmacia Biotech, Les Ulis, France). Semi-quantitative analysis was carried out based on the optical density (OD) of scanned films (Quantity One 1-D image analysis software version 4.4; Biorad, Hercules, CA, USA). Specific ODs were normalized with respect to those for tubulin for experiments on total homogenates. The specific OD was then normalized with respect to the amount of tubulin loaded in the corresponding lane of the same gel. A partition ratio was calculated and expressed as a percentage.

#### RT-PCR analysis

Total RNA was extracted, 48 hours after transfection, with Trizol reagent (Invitrogen, Cergy Pontoise, France). Real-time quantitative RT-PCR was performed in triplicate, with 0.4% random-primed cDNAs generated from 400 ng total RNA. PCR was carried out in a 20 µl reaction volume containing Platinium SYBR Green pPCR super Mix-UDG (Invitrogen, Cergy Pontoise, France), and 10 µM of both forward (HATAX-1F: GGCTCACTTTGTGCTCAACATTG) and reverse (HATAX-2R: TCTCATCCTCTCCTCCTCATCCAG) primers. An ABI PRISM 7000 thermal cycler was programmed for an initial denaturation step (95°C, 2 min) followed by 40 amplification cycles (95°C, 15 s; 60°C, 1 min). The amplification rate for each target was evaluated from the cycle threshold (Ct) numbers obtained with cDNA dilutions, with correction for human β-actin levels (B-ACTIN-1F: TGAAGGTGACAGCAGTCGGTTG; B-ACTIN-2R:GGCTTTTAGGATGGCAAGGGAC), which were assumed to be constant. Differences between control and experimental samples were calculated using the 2^−ΔΔCt^ method [41]. *LacZ* oligos were used as an internal standard for evaluating transfection efficiency (LacZ-1F: CCTTACTGCCGCCTGTTTTGAC; LacZ-2R: TGATGTTGAACTGGAAGTCGCC). RT-PCR analysis was performed on 3 to 5 samples from 3 independent transfections. Data are expressed as the mean of normalized values, as relative ATX3 mRNA level±SEM. Statistical analysis was performed by one-way analysis of variance (ANOVA) followed by Fisher's PLSD *post-hoc* test (StatView 4.0, version 3.2.6; Aladdin Systems). Values of p<0.05 were considered significant.

### *In vivo* experiments

#### Animals

Adult male Wistar rats (Iffa Credo/Charles River, Les Oncins, France), each weighing ∼200 g were used. The animals were housed in a temperature-controlled room maintained on a 12 h light/12 h dark cycle. Food and water were provided *ad libitum.* The experiments were carried out in accordance with the European Community Council directive (86/609/EEC) for the care and use of laboratory animals.

### *In vivo* injection of lentiviral vectors

Concentrated viral stocks were thawed on ice and resuspended by repeated pipetting. The rats were anesthetized as previously described [36]. Lentiviral vectors encoding human mutant ataxin-3(C) and shRNA (shAtaxMUT(C), shAtaxWT(G), or shGFP) were co-injected into the rat striatum. Viral particle content was adjusted to 200,000 ng of p24/ml. The animals received a single 5 µl injection containing both lentiviruses into each side, at the following coordinates: 0.5 mm rostral to bregma, ±3 mm lateral to midline, and 5 mm ventral to the skull surface, with the mouth bar set at 0. The viral suspensions were injected at a rate of 0.2 µl/min through an automatic injector (Stoelting Co., Wood Dale, USA), the needle being left in place for an additional 5 minutes. The skin was closed using wound clips (Phymep, Paris, France).

### Histological processing

#### Tissue preparation

Three (n = 2 for shAtaxMUT and shAtaxWT) or 8 weeks (n = 7 for shAtaxMUT, n = 8 for shAtaxWT and n = 4 for shGFP) after lentivirus injection, the animals were perfused with a phosphate solution followed by fixation with 4% paraphormaldehide (PAF, Fluka, Sigma, Buchs, Switzerland) and 10% picric acid, their brains removed and the entire striatum was sliced, the sections then being stored in 48-well trays (Corning Inc., NY, USA), as previously described [37].

### Immunohistochemical procedure

The immunohistochemical procedure was initiated by endogenous peroxidase quenching by incubation for 1 h at 37°C in 0.1% diphenylhydrazine in PBS. Free-floating sections were incubated for 1 h at room temperature in 0.1% Triton X-100 in PBS (or 0.02% Triton X-100 in PBS for the anti-ubiquitin antibody) supplemented with 10% NGS (normal goat serum, Gibco), and then with the appropriate antibodies—the mouse monoclonal anti-ataxin-3 antibody 1H9 (1∶5000; overnight, 4°C), a rabbit polyclonal anti-ataxin-3 antibody (1∶2000, overnight, 4°C), kindly provided by Dr. Henry L. Paulson (University of Iowa, USA), a rabbit polyclonal anti-ubiquitin antibody (Dakocytomation, Zug, Switzerland; 1∶1000; O/N 4°C), a rabbit polyclonal dopamine- and cAMP-regulated phosphoprotein with a molecular mass of 32 kDa (DARPP-32; Chemicon,Temecula, CA, USA; 1∶5000, overnight, 4°C) and an anti-β-galactosidase antibody (1∶4000; Chemicon, Temecula, CA,USA) diluted in 0.1% Triton X-100 in PBS supplemented with 10% NGS. Sections were washed three times and incubated with the corresponding biotinylated secondary antibody (1∶200; Vector Laboratories Inc, CA, USA) diluted in 0.1% Triton X-100 in PBS supplemented with 10% NGS for 2 h at room temperature. Sections were washed three times and bound antibodies were visualized with the ABC amplification system (Vectastain ABC kit, Vector Laboratories, West Grove, USA) and 3,3′-diaminobenzidine tetrahydrochloride (peroxidase substrate kit DAB; Vector Laboratories, CA, USA) as the substrate. The sections were mounted, dehydrated by two passages through ethanol and toluol solutions, and coverslipped with Eukitt® (O. Kindler GmbH & CO. Freiburg, Germany).

Double-staining for ataxin-3/DARPP-32 and ataxin-3/β-galactosidase - Free-floating sections were incubated at room temperature for 1 h in 0.1% Triton X-100 in PBS supplemented with 10% NGS (normal goat serum, Gibco), followed by blocking solution containing the corresponding antibodies: 1H9 Ab (1∶5000) and anti-DARPP-32 (1∶2000) overnight, 4°C; 1H9 (1∶5000) and anti-β-galactosidase (rabbit polyclonal antibody; Chemicon, Temecula, CA, USA, 1∶4000, overnight, 4°C). Sections were washed three times and incubated with the corresponding secondary antibodies coupled to fluorophores (1∶200; Molecular Probes, Oregon, USA) diluted in 0.1% Triton X-100 in PBS supplemented with 10% NGS for 2 h at room temperature. The sections were washed three times in PBS and mounted in Fluorsave Reagent (Calbiochem, Germany) on microscope slides.

### Evaluation of the volume of the DARPP-32-depleted region

The extent of ataxin-3 lesions in the striatum was analyzed by digitizing 10 DARPP-32-stained sections per animal (25 µm sections at 200 µm intervals), selected so as to obtain complete rostrocaudal sampling of the striatum with a slide scanner and by quantifying the area of the lesion with a semiautomated image-analysis program (Image J, USA). Sections from throughout the entire striatum were analyzed. The area of the striatum showing a loss of DARPP-32 staining was measured for each animal, with an operator-independent macro. The volume was then estimated with the following formula: volume  = *d*(*a*_1_+*a*_2_+*a*_3_ …), where *d* is distance between serial sections (200 µm or 300 µm), and *a*_1_, *a*_2_, *a*_3_ .are DARPP-32-depleted areas for individual serial sections. The depleted area corresponds to area with a gray-scale value lower than the mean gray-scale value of all pixels measured in the lesioned area. Data are expressed as the area of the evaluated DARPP-32-depleted region.

### Cell counts and morphometric analysis of ataxin-3 inclusions

Cell counts and morphometric analysis of ataxin-3 inclusions were performed as previously described, but with minor modifications [38], [39]. Coronal sections showing complete rostrocaudal sampling (1 of 12 sections or 1 of 8 sections) of the striatum were scanned with a ×10 objective, using a Zeiss (Oberkochen, Germany) Axioplan 2 imaging microscope motorized for X, Y, and Z displacements and an image acquisition and analysis system (Morphostar V 6.0; IMSTAR, Paris, France). The analyzed areas of the striatum encompassed the entire region containing mutant ATX3 aggregates, as revealed by staining with the anti-ataxin-3 antibody. This area corresponded, on average, to 100 contiguous digitized images per section. Image pixels were 0.8 µm×0.8 µm in size. Section lighting was similar for all acquisitions and was automatically corrected using blank images. Images were automatically segmented for the quantification of dark objects (aggregates/inclusions), using the same parameters defining light intensity threshold, size and shape object filters. With this procedure, all inclusions with an apparent cross-sectional area greater than 3 µm^2^ were reliably detected. None of the objects touching one of the X or Y borders of the fields of view were counted, in any of the images. For each animal, the estimated total number of inclusions (Ne) was calculated as Ne = (Ns)/Sf, where Ns is the number of inclusions detected in all sections and Sf is the rostrocaudal sampling fraction (1/8). Inclusions in striatal neurons tended to be round (mean rotundity index>0.90), with an isotropic orientation in the striatum. We therefore corrected the number of raw cell counts using the Abercrombie factor [40]. This factor was calculated using the formula, A = N/N+h where N is section thickness and h is mean object height, estimated for each experimental group based on morphometric analysis of segmented objects (shGFP, 6.61 µm; shAtaxWT(G), 6.51 µm; shAtaxMUT(C), 6.18 µm). The corrected total number of inclusions (Nc) was calculated as Nc = A×Ne.

#### Cresyl violet staining

Premounted sections were stained with cresyl violet for 2 minutes, differentiated in acetate buffer pH 3.8 to 4 (2.72% sodium acetate and 1.2% acetic acid; 1∶4 v/v), dehydrated by passing twice through ethanol and toluol solutions, and mounted on microscope slides with Eukitt® (O. Kindler GmbH & CO. Freiburg, Germany).

#### Flurojade B staining

We stained striatal sections with FluoroJade-B (Chemicon, Temecula, CA), an anionic fluorescein derivative that stains neurons undergoing degeneration. The sections were first washed in water and then mounted on silane-coated glass slides, dehydrated, and stained according to the supplier's manual.. Bright-field and fluorescent images were acquired digitally on an Axioskop 2 Plus microscope (Zeiss) with Axiovision software version 4.2. All photographs for comparison were taken under identical image acquisition conditions and uniform adjustments of brightness and contrast were made to all images.

### Data analysis

The intensity of immunostaining is expressed as mean±SEM. Statistical analysis was performed by one-way analysis of variance (ANOVA) followed by Fisher's PLSD *post hoc* test (StatView 4.0, version 3.2.6; Aladdin Systems). Values of *p*<0.05 were considered statistically significant.

## References

[1] Sudarsky L, Coutinho P (1995). Machado-Joseph disease.. Clin Neurosci.

[2] Gwinn-Hardy K, Singleton A, O'Suilleabhain P, Boss M, Nicholl D (2001). Spinocerebellar ataxia type 3 phenotypically resembling parkinson disease in a black family.. Arch Neurol.

[3] Wullner U, Reimold M, Abele M, Burk K, Minnerop M (2005). Dopamine transporter positron emission tomography in spinocerebellar ataxias type 1, 2, 3, and 6.. Arch Neurol.

[4] Alves S, Regulier E, Nascimento-Ferreira I, Hassig R, Dufour N (2008). Striatal and nigral pathology in a lentiviral rat model of Machado-Joseph disease.. Hum Mol Genet.

[5] Ranum LP, Lundgren JK, Schut LJ, Ahrens MJ, Perlman S (1995). Spinocerebellar ataxia type 1 and Machado-Joseph disease: incidence of CAG expansions among adult-onset ataxia patients from 311 families with dominant, recessive, or sporadic ataxia.. Am J Hum Genet.

[6] Kawaguchi Y, Okamoto T, Taniwaki M, Aizawa M, Inoue M (1994). CAG expansions in a novel gene for Machado-Joseph disease at chromosome 14q32.1.. Nat Genet.

[7] Cancel G, Abbas N, Stevanin G, Durr A, Chneiweiss H (1995). Marked phenotypic heterogeneity associated with expansion of a CAG repeat sequence at the spinocerebellar ataxia 3/Machado-Joseph disease locus.. Am J Hum Genet.

[8] Maruyama H, Nakamura S, Matsuyama Z, Sakai T, Doyu M (1995). Molecular features of the CAG repeats and clinical manifestation of Machado-Joseph disease.. Hum Mol Genet.

[9] Schmidt T, Landwehrmeyer GB, Schmitt I, Trottier Y, Auburger G (1998). An isoform of ataxin-3 accumulates in the nucleus of neuronal cells in affected brain regions of SCA3 patients.. Brain Pathol.

[10] Harper SQ, Staber PD, He X, Eliason SL, Martins IH (2005). RNA interference improves motor and neuropathological abnormalities in a Huntington's disease mouse model.. Proc Natl Acad Sci U S A.

[11] Rodriguez-Lebron E, Denovan-Wright EM, Nash K, Lewin AS, Mandel RJ (2005). Intrastriatal rAAV-mediated delivery of anti-huntingtin shRNAs induces partial reversal of disease progression in R6/1 Huntington's disease transgenic mice.. Mol Ther.

[12] DiFiglia M, Sena-Esteves M, Chase K, Sapp E, Pfister E (2007). Therapeutic silencing of mutant huntingtin with siRNA attenuates striatal and cortical neuropathology and behavioral deficits.. Proc Natl Acad Sci U S A.

[13] Raoul C, Abbas-Terki T, Bensadoun JC, Guillot S, Haase G (2005). Lentiviral-mediated silencing of SOD1 through RNA interference retards disease onset and progression in a mouse model of ALS.. Nat Med.

[14] Ralph GS, Radcliffe PA, Day DM, Carthy JM, Leroux MA (2005). Silencing mutant SOD1 using RNAi protects against neurodegeneration and extends survival in an ALS model.. Nat Med.

[15] Xia H, Mao Q, Eliason SL, Harper SQ, Martins IH (2004). RNAi suppresses polyglutamine-induced neurodegeneration in a model of spinocerebellar ataxia.. Nat Med.

[16] Doss-Pepe EW, Stenroos ES, Johnson WG, Madura K (2003). Ataxin-3 interactions with rad23 and valosin-containing protein and its associations with ubiquitin chains and the proteasome are consistent with a role in ubiquitin-mediated proteolysis.. Mol Cell Biol.

[17] Miller VM, Xia H, Marrs GL, Gouvion CM, Lee G (2003). Allele-specific silencing of dominant disease genes.. Proc Natl Acad Sci U S A.

[18] Stevanin G, Cancel G, Didierjean O, Durr A, Abbas N (1995). Linkage disequilibrium at the Machado-Joseph disease/spinal cerebellar ataxia 3 locus: evidence for a common founder effect in French and Portuguese-Brazilian families as well as a second ancestral Portuguese-Azorean mutation.. Am J Hum Genet.

[19] Gaspar C, Lopes-Cendes I, DeStefano AL, Maciel P, Silveira I (1996). Linkage disequilibrium analysis in Machado-Joseph disease patients of different ethnic origins.. Hum Genet.

[20] Gaspar C, Lopes-Cendes I, Hayes S, Goto J, Arvidsson K (2001). Ancestral origins of the Machado-Joseph disease mutation: a worldwide haplotype study.. Am J Hum Genet.

[21] Gonzalez-Alegre P, Miller VM, Davidson BL, Paulson HL (2003). Toward therapy for DYT1 dystonia: allele-specific silencing of mutant TorsinA.. Ann Neurol.

[22] Xia X, Zhou H, Huang Y, Xu Z (2006). Allele-specific RNAi selectively silences mutant SOD1 and achieves significant therapeutic benefit in vivo.. Neurobiol Dis.

[23] Matsumura R, Takayanagi T, Murata K, Futamura N, Hirano M (1996). Relationship of (CAG)nC configuration to repeat instability of the Machado-Joseph disease gene.. Hum Genet.

[24] Reynolds A, Leake D, Boese Q, Scaringe S, Marshall WS (2004). Rational siRNA design for RNA interference.. Nat Biotechnol.

[25] Chai Y, Berke SS, Cohen RE, Paulson HL (2004). Poly-ubiquitin binding by the polyglutamine disease protein ataxin-3 links its normal function to protein surveillance pathways.. J Biol Chem.

[26] Berke SJ, Chai Y, Marrs GL, Wen H, Paulson HL (2005). Defining the role of ubiquitin-interacting motifs in the polyglutamine disease protein, ataxin-3.. J Biol Chem.

[27] Boeddrich A, Gaumer S, Haacke A, Tzvetkov N, Albrecht M (2006). An arginine/lysine-rich motif is crucial for VCP/p97-mediated modulation of ataxin-3 fibrillogenesis.. Embo J.

[28] Macedo-Ribeiro S, Pereira de Almeida L, Carvalho A, Rego A, Malva J, Rego A, Oliveira C, Cunha R (2007). Polyglutamine expansion diseases – the case of Machado-Joseph disease.. Interactions Between Neurons and Glia in Aging and Disease Springer.

[29] Schmitt I, Linden M, Khazneh H, Evert BO, Breuer P (2007). Inactivation of the mouse Atxn3 (ataxin-3) gene increases protein ubiquitination.. Biochem Biophys Res Commun.

[30] Rodrigues AJ, Coppola G, Santos C, Costa Mdo C, Ailion M (2007). Functional genomics and biochemical characterization of the C. elegans orthologue of the Machado-Joseph disease protein ataxin-3.. Faseb J.

[31] Warrick JM, Morabito LM, Bilen J, Gordesky-Gold B, Faust LZ (2005). Ataxin-3 suppresses polyglutamine neurodegeneration in Drosophila by a ubiquitin-associated mechanism.. Mol Cell.

[32] Sobue G, Doyu M, Nakao N, Shimada N, Mitsuma T (1996). Homozygosity for Machado-Joseph disease gene enhances phenotypic severity.. J Neurol Neurosurg Psychiatry.

[33] Lang AE, Rogaeva EA, Tsuda T, Hutterer J, St George-Hyslop P (1994). Homozygous inheritance of the Machado-Joseph disease gene.. Ann Neurol.

[34] Li LB, Yu Z, Teng X, Bonini NM (2008). RNA toxicity is a component of ataxin-3 degeneration in Drosophila.. Nature.

[35] Hottinger AF, Azzouz M, Deglon N, Aebischer P, Zurn AD (2000). Complete and long-term rescue of lesioned adult motoneurons by lentiviral-mediated expression of glial cell line-derived neurotrophic factor in the facial nucleus.. J Neurosci.

[36] Regulier E, Trottier Y, Perrin V, Aebischer P, Deglon N (2003). Early and reversible neuropathology induced by tetracycline-regulated lentiviral overexpression of mutant huntingtin in rat striatum.. Hum Mol Genet.

[37] de Almeida LP, Ross CA, Zala D, Aebischer P, Deglon N (2002). Lentiviral-mediated delivery of mutant huntingtin in the striatum of rats induces a selective neuropathology modulated by polyglutamine repeat size, huntingtin expression levels, and protein length.. J Neurosci.

[38] Palfi S, Brouillet E, Jarraya B, Bloch J, Jan C (2007). Expression of mutated huntingtin fragment in the putamen is sufficient to produce abnormal movement in non-human primates.. Mol Ther.

[39] Arango M, Holbert S, Zala D, Brouillet E, Pearson J (2006). CA150 expression delays striatal cell death in overexpression and knock-in conditions for mutant huntingtin neurotoxicity.. J Neurosci.

[40] Clarke PG (1992). How inaccurate is the Abercrombie correction factor for cell counts?. Trends Neurosci.

[41] Livak KJ, Schmittgen TD (2001). Analysis of relative gene expression data using real-time quantitative PCR and the 2(-Delta Delta C(T)) Method.. Methods.

